# Research on the new mechanism and path of delayed retirement affecting the birth rate

**DOI:** 10.3389/fpubh.2025.1660541

**Published:** 2025-09-15

**Authors:** Zhang Jinmeng, Guofeng Guan, Jie Guan

**Affiliations:** ^1^School of Economics, Xinjiang College of Science and Technology, Korla, China; ^2^School of Science, Hong Kong Baptist University, Hong Kong, China

**Keywords:** delayed retirement, grandparental caregiving, the birth rate, OLG model, social welfare

## Abstract

This paper incorporates time resource spent on caregiving, economic resource spent on caregiving, and delayed retirement into an overlapping generations (OLG) model to examine how delayed retirement affects the birth rate and through which pathways. It further measures and analyzes the impact of delayed retirement on the proportion of economic resource spent on caregiving relative to the total economic cost of raising children, as well as the joint effects on social welfare. The findings show that delayed retirement leads to a decline in the birth rate. Although it increases older participation in economic resource spent on caregiving, the positive effect of raising its proportion in the total cost of childrearing is outweighed by the negative effect of reduced time resource spent on caregiving. Numerical simulations reveal a reverse relationship between time resource spent on caregiving and economic resource spent on caregiving under the maximization of social welfare. Delayed retirement increases older participation in economic resource spent on caregiving and improves social welfare to some extent. Overall, social welfare rises because the positive effect of increased economic support outweighs the negative effect associated with delayed retirement. Therefore, it is necessary to promote the gradual implementation of delayed retirement and complement it with measures that reduce the time cost of childrearing for young parents so as to support higher fertility.

## 1 Introduction

Population aging is a challenge faced by many countries, and China is no exception. Data from the seventh census show that more than 264 million people in China are aged 60 or above, accounting for 18.7% of the total population. According to an international benchmark, a country is considered aging if people aged 60 and above account for at least 10% of the total population. The proportion of the older population over the age of 60 in China is 18.7%, far exceeding the defining standard of 10%, China has entered an aging society and the aging degree is deepening, the decline in the birth rate is an important cause of aging. China’s birth rate and growth rate of population began to plummet in 2016. The birth rate even fell below 1‰ in 2020 and 2021, and the population growth rate in 2021 dropped below 1‰ for the first time since 1961. Low fertility will further aggravate population aging. Aging will bring a series of problems. An excessively large older population reduces the labor force and lowers productivity. It also affects the sustainability of pensions. For example, due to the aging population, the Dutch pension expenditure is much higher than the tax revenue, which seriously endangers the sustainability of the country’s pension.

To address the negative impact of population aging on the economy and pension sustainability, many countries have implemented delayed retirement policies. For example, the United Kingdom has adopted a series of pension bills to dynamically adjust the retirement age, the United States has implemented a flexible retirement system within the range of 62–70 years old, and Japan has phased in the delayed retirement policy, and so on. China put forward the concept of delayed retirement at the Third Plenum of the 18th CPC Central Committee in 2013. The exact policy is yet to be decided because the implementation of the delayed retirement policy and the setting of the scheme are controversial, but the implementation of the delayed retirement policy is imperative. The report of the 20th National Congress further pointed out that the statutory retirement age should be gradually raised. In 2022, Shandong Province and Jiangsu Province officially began to implement the gradual retirement delay policy as pilots. On the other hand, China has also actively adjusted its childbearing policy in an attempt to cope with the urgent aging situation by increasing the birth rate. However, the adjustment of the birth policy cannot reverse the general trend of declining birth rate.

Nowadays, the pressure on young people’s lives is increasing, forcing them to devote more time and energy to work, so the older individuals become the providers of grandchildren care resources. Feng and Han (1) found that about 30% of the retired older individuals provide grandparental caregiving, indicating that grandparental caregiving has become a common phenomenon and plays an important role in the upbringing of grandchildren. Delayed retirement policies extend working time for old people will reduce the amount of time they can provide for grandparental caregiving. When the older individuals cannot provide caring resources in terms of time, they will make economic compensation that is increasing their participation in economic grandparental rearing. Then will the implementation of the retirement delay policy affect the birth rate and how? This is a question worth thinking.

As a short-term tool to alleviate the pressure of population aging, delayed retirement has attracted extensive scholarly attention both domestically and internationally. Most studies examine how retirement age adjustments affect the socio-economic system and individual well-being. Galasso and Profeta (2) showed that delayed retirement age can prevent further increases in the pension contribution rate. At the same time, to a certain extent, it can alleviate the pressure on pension payments brought by the continuous development of the aging population and enhance the sustainable payment ability of pensions (3). Bovenberg (4) considered that delaying the valid retirement age is a powerful tool to maintain the stable financing of the pension policy in the aging society, and can take into account the overall interests of retired workers and society simultaneously (5). Kalwij and Kapteyn (6) found that the delayed retirement policy can improve the pension welfare of the older individuals because the delayed retirement policy will increase the average working age of the whole society and improve the total factor productivity (7). Belan et al. (8) took the household savings rate as a measure of welfare and found that the delayed retirement policy would reduce the welfare of households. Cabo and García-González (9) pointed out that delayed retirement prevents the Social Security deficit from increasing to the unbounded public debt level. Martín et al. (10) found that increasing the retirement age can enhance social demand and promote employment (11–13). Chen (14) found that there were obvious differences in the impact of delayed retirement policies among different groups, and the low-income groups were the most negatively affected.

With the decline in the birth rate, the impact of delayed retirement on the birth rate has gradually attracted the attention of scholars. Time resource spent on caregiving and economic resource spent on caregiving played an important role. Time resource spent on caregiving refers to the time spent by grandparents taking care of their grandchildren, abbreviated as TRS in this paper. Economic resource spent on caregiving refers to the expenses spent by grandparents taking care of their grandchildren, abbreviated as ERS in this paper. And the proportion of economic resource spent on caregiving to the total economic cost of raising children refers to the proportion of economic support provided by grandparents to their grandchildren in the total economic cost of their growth, abbreviated as PERS in this paper. Yan (15) found that the delayed retirement policy will increase income in the old period, thereby reducing the pressure to save money when young, who tend to devote less time to labor and more time to raising children. At the same time, the delayed retirement policy will also reduce the TRS and reduce the birth rate (16, 17). Du and Lin (18) considered that the delayed retirement policy can only alleviate the decline rate of the population birth rate to a certain extent, and the effect on the increased population birth rate cannot be sustainable. The above studies were all researched at the theoretical level. Gu et al. (19) used the CHNS data to study empirically and found that the delayed retirement policy would reduce the birth rate, and the birth rate is negatively affected mainly by the TRS. From the perspective of endowment burden, Chen et al. (20) further found that the impact of delayed retirement on the birth rate varies with the change of pension payment patterns, and the delayed retirement policy has different impacts on the birth rate under different pension payment patterns (21).

The existing literature exploration of the impact of delayed retirement on the birth rate is mostly based on the overlapping generation model (OLG model), which is studied through the TRS system. However, this paper believes that the older individuals can provide TRS as well as ERS, and ERS also has an important impact on the birth rate. Existing studies only consider that the TRS system will underestimate the impact of delayed retirement on the birth rate to a certain extent. Therefore, this paper establishes the OLG model including TRS and PERS, delayed retirement, and the birth rate to study the new mechanism of delayed retirement affecting the birth rate. At the same time, the impact of delayed retirement on social welfare under the new mechanism will be discussed. This will provide relevant theoretical support for Chinese policy formulation and has strong practical significance.

## 2 Construction of OLG model

### 2.1 Individual

Representative individuals in society include the young period and old period, and the representative individuals have 1 unit of time endowment in both the young period and old period. At a young period, the time endowment is used to take care of offspring and work, and the income is used to pay pension insurance, consume, save, and raise offspring. Under delayed retirement, individuals allocate their time endowment in old age to continued work, TRS, and leisure. In old age, they finance consumption and economic resource spent on caregiving using pension benefits, the present value of savings from youth, and wage income earned while working.

The utility of the life of a representative individual is derived from consumption in the young period and old period, as well as the number of children in youth. Consumption in the young period $C_{1,t}$ , consumption in the old period $C_{2,t+1}$ , and total utility level U can be expressed as:


(1) $$C_{1,t}+S_{t}=(1-\tau )w_{t}[1-({\mathrm{vn}}_{t}-\frac{z_{t}}{n_{t-1}})]-w_{t}(1-\delta ){\mathrm{\mu n}}_{t}$$

(2) $$C_{2,t+1}=R_{t+1}S_{t}+(1-\tau )xw_{t+1}+(1-x)P_{t+1}-{\mathrm{\delta \mu n}}_{t}w_{t}$$

(3) $$\max U=\max[\ln C_{1,t}+\beta \ln C_{2,t+1}+\theta \ln n_{t}]$$

In Equation 1, $S_{t}$ refers to savings, $w_{t}$ represents the salary level in the period t , τ is the social security contribution rate, and $n_{t}$ is the number of children or the birth rate. This paper introduces the time cost of raising children, v represents the time investment of individuals raising unit offspring at a young period, and ${\mathrm{vn}}_{t}$ refers to the total time spent by individuals raising children in the young period. $z_{t}$ represents the TRS in the period t and the total number of older people in the period t is $L_{t-1}$ , so the total TRS is $z_{t}L_{t-1}$ . The total number of young people in the period t is $L_{t}$ . By the population growth equation $L_{t}/L_{t-1}=n_{t-1}$ , each young person receives TRS equal to $z_{t}L_{t-1}/L_{t}=z_{t}/n_{t-1}$ . Hence, ${\mathrm{vn}}_{t}-\frac{z_{t}}{n_{t-1}}$ represents the total time that young people need to devote to childrearing after receiving TRS. μ is the proportion that the cost of raising each child accounts for wages, δ is the PERS, and measures the ERS accounts for the total economic cost of raising children.

In Equation 2, $R_{t+1}$ is the interest rate, x is working time in old age, and its magnitude reflects the duration of delayed retirement (additional years worked beyond the baseline retirement age). The larger the value is, the later the retirement time will be. $(1-\tau )xw_{t+1}$ is the salary earned in the old period. $P_{t+1}$ represents the level of pension received after retirement, $(1-x)P_{t+1}$ represents the total social security income received after retirement, and ${\mathrm{\delta \mu n}}_{t}w_{t}$ represents the total PERS. In Equation 3, β is the utility discount factor in the old period, and θ is the preference degree for the number of children.

From Equations 1, 2, we can get the relationship between lifetime income and expenditure of representative individuals.


(4) $$\begin{array}{l} C_{1,t}+\frac{C_{2,t+1}}{R_{t+1}}=(1-\tau )w_{t}[1-({\mathrm{vn}}_{t}-\frac{z_{t}}{n_{t-1}})]-w_{t}(1-\delta ){\mathrm{\mu n}}_{t}\\ +\frac{(1-\tau )xw_{t+1}+(1-x)P_{t+1}-{\mathrm{\delta \mu n}}_{t}w_{t}}{R_{t+1}} \end{array}$$

Under the restriction of Equation 4, representative individuals maximize their own utility by choosing savings and the number of offspring. By constructing the Lagrange Function to solve the maximizing first-order conditions for the total utility Equation 3, we can get (Equations 5, 6 and 7).


(5) $$C_{2,t+1}=\beta R_{t+1}C_{1,t}$$

(6) $$\begin{array}{l} S_{t}=(1-\tau )w_{t}[1-({\mathrm{vn}}_{t}-\frac{z_{t}}{n_{t-1}})]-w_{t}(1-\delta ){\mathrm{\mu n}}_{t}-\\ \frac{(1-\tau )w_{t}[1-({\mathrm{vn}}_{t}-\frac{z_{t}}{n_{t-1}})]-w_{t}(1-\delta ){\mathrm{\mu n}}_{t}+\frac{(1-\tau )xw_{t+1}+(1-x)P_{t+1}-{\mathrm{\delta \mu n}}_{t}w_{t}}{R_{t+1}}}{1+\beta } \end{array}$$

(7) $$\frac{\theta }{n_{t}}C_{1,t}=(1-\tau )w_{t}v+w_{t}(1-\delta )\mu +\frac{{\mathrm{\delta \mu w}}_{t}}{R_{t+1}}$$

### 2.2 Enterprise

It is assumed that the enterprise is in a perfectly competitive market and inputs capital and labor to get output. The production function as shown in Equation 8:


(8) $$Y_{t}=AK_{t}^{\alpha }N_{t}^{1-\alpha }$$

Among them, A is the level of comprehensive technology, $K_{t}$ is the level of capital invested in the enterprise, $N_{t}=[1-({\mathrm{vn}}_{t}-\frac{z_{t}}{n_{t-1}})]L_{t}+xL_{t-1}$ represents the total labor force in the economy, and 0<α<1 represents the capital output elasticity. This paper defines $k_{t}=K_{t}/N_{t}$ as capital per worker.

It is assumed that capital is fully depreciated in a period, the enterprise maximizes its profit by selecting capital and labor. By solving the profit maximization problem, we can get:


(9) $$R_{t}=\mathrm{A\alpha}k_{t}^{\alpha -1}$$

(10) $$w_{t}=A(1-\alpha )k_{t}^{\alpha }$$

### 2.3 Social security

Suppose that under the pay-as-you-go social security system, the social security fund paid by the young people is allocated to the current older people, and the equation of fund income and expenditure is as follows:


(11) $$w_{t+1}\tau [1-(vn_{t+1}-\frac{z_{t+1}}{n_{t}})]L_{t+1}+\tau w_{t+1}{\mathrm{xL}}_{t}=(1-x)P_{t+1}L_{t}$$

The left side of the equation is the total income of the current period social security fund, $w_{t+1}\tau [1-(vn_{t+1}-\frac{z_{t+1}}{n_{t}})]L_{t+1}$ is the social security fund paid by the young people during the current period, and $\tau w_{t+1}{\mathrm{xL}}_{t}$ is the social security fund paid by the old people who delay retirement. The right side of the equation is the total expenditure of the social security fund. Divide both sides of Equation 11 by $L_{t}$ to get Equation 12:


(12) $$w_{t+1}\tau [1-(vn_{t+1}-\frac{z_{t+1}}{n_{t}})]n_{t}+w_{t+1}\mathrm{x\tau}=(1-x)P_{t+1}$$

### 2.4 Capital market

Assuming that the capital market clears, the capital accumulation in the current period comes from the savings of representative individuals and is all used for the capital investment in the next period. The dynamic equation of capital accumulation is as follows:


(13) $$K_{t+1}=S_{t}L_{t}$$

Divide both sides of Equation 13 by the total labor $N_{t+1}$ of period t+1 to obtain the capital per worker $k_{t+1}$ of period t+1 :


(14) $$\begin{array}{l} k_{t+1}=\frac{K_{t+1}}{[1-(vn_{t+1}-\frac{z_{t+1}}{n_{t}})]L_{t+1}+{\mathrm{xL}}_{t}}=\\ \;\;\;\;\;\;\;\;\;\;\;\;\;\;\;\frac{S_{t}}{[1-(vn_{t+1}-\frac{z_{t+1}}{n_{t}})]n_{t}+x} \end{array}$$

### 2.5 General equilibrium

The production function in this paper conforms to the neoclassical growth framework and does not consider technological progress. When the economy converges to the equilibrium state, it satisfies: $k_{t+1}=k_{t}=k^{*}$ _,_ $n_{t+1}=n_{t}=n^{*}$ , and substituting the above conditions into (7, 14), we can get:


(15) $$\begin{array}{l} {\left(k^{*}\right)}^{\alpha -1}=\\ \frac{\delta \mu (1+\beta +\theta )-n^{*}\theta [x+\tau (n^{*}-{\left(n^{*}\right)}^{2}v+z)]}{\mathrm{A\alpha}({\left(n^{*}\right)}^{2}+{\left(n^{*}\right)}^{2}\beta +{\left(n^{*}\right)}^{2}\theta )[\mu (\delta -1)+v(\tau -1)]-\mathrm{A\alpha}(n^{*}+z)\theta (\tau -1)} \end{array}$$

(16) $$\begin{array}{l} {\left(k^{*}\right)}^{\alpha -1}=\\ \frac{\mathrm{v\tau}{\left(n^{*}\right)}^{3}-n^{*}x-\mathrm{z\tau}n^{*}-{\left(n^{*}\right)}^{2}(\tau -\mu \delta )-\frac{\alpha n^{*}(n^{*}-{\left(n^{*}\right)}^{2}v+x+z)(1+\beta )}{1-\alpha }}{\text{Aαzβ}(\tau -1)+\mathrm{A\alpha}n^{*}\beta (\tau -1)+\mathrm{A\alpha \beta}{\left(n^{*}\right)}^{2}(v+\mu -\mu \delta -\mathrm{v\tau})} \end{array}$$

To study how delayed retirement and PERS affect the equilibrium birth rate, define the right-hand sides of Equations 15,16 as $\psi (x,\delta ,n^{*})$ and $\varpi (x,\delta ,n^{*})$ , respectively, $\Omega (x,\delta ,n^{*})=\varpi (x,\delta ,n^{*})-\psi (x,\delta ,n^{*})=0$ . By the implicit function theorem, we obtain Equations 17,18:


(17) $$\frac{dn^{*}}{\mathrm{dx}}=-\frac{\Omega_{x}(x,\delta ,n^{*})}{\Omega_{n^{*}}(x,\delta ,n^{*})}=-\frac{\varpi_{x}(x,\delta ,n^{*})-\psi_{x}(x,\delta ,n^{*})}{\varpi_{n^{*}}(x,\delta ,n^{*})-\psi_{n^{*}}(x,\delta ,n^{*})}$$

(18) $$\frac{dn^{*}}{\mathrm{d\delta}}=-\frac{\Omega_{\delta }(x,\delta ,n^{*})}{\Omega_{n^{*}}(x,\delta ,n^{*})}=-\frac{\varpi_{\delta }(x,\delta ,n^{*})-\psi_{\delta }(x,\delta ,n^{*})}{\varpi_{n^{*}}(x,\delta ,n^{*})-\psi_{n^{*}}(x,\delta ,n^{*})}$$

Through calculation, $\mathrm{sign}(dn^{*}/\mathrm{d\delta})>0$ shows that the increase in PERS will increase the birth rate in the equilibrium state. This is because the increase in PERS will reduce the economic cost of raising children for young people, increasing the birth rate. On the other hand, the increase in PERS has a negative effect on the capital per worker in equilibrium, leading to a decline in the wage level of young people, young people need to spend more time working in order to meet the daily consumption and savings, resulting in a decline in the birth rate. Under the framework of this paper, the positive effect of increasing the PERS on the birth rate is greater than the negative effect, so the birth rate increases.

In the same way, $\mathrm{sign}(dn^{*}/\mathrm{dx})<0$ , that is, delayed retirement will reduce the birth rate in the equilibrium state. The impact of delayed retirement on the birth rate has the following two mechanisms: On the one hand, delayed retirement will reduce the TRS, and young people will need to spend more time raising their children, resulting in a decline in the birth rate; On the other hand, delayed retirement will increase the PERS, which will increase the birth rate. Under the framework of this paper, the positive effect of increasing the PERS on the birth rate is smaller than the negative effect of the TRS so the delayed retirement policy can have a negative effect on the birth rate.

## 3 Optimal PERS

The above conclusions are results calculated under the condition of equilibrium among all sectors of society. In order to obtain the optimal PERS, a social welfare function should be introduced. In this paper, the social welfare function is defined as the utility-weighted sum of typical individuals of all generations, and the social welfare function is defined as follows:


(19) $$W=\rho \beta C_{2,0}+\sum_{t=1}^{\infty }\rho^{t-1}[\ln C_{1,t}+\beta C_{2,t+1}+\theta \ln n_{t}]$$

Among them, W is the sum of the weighted utility of representative individuals of each generation from now on, and ρ is the social discount factor, reflecting the government’s emphasis on the utility of each generation. The meanings of other variables and parameters are the same as those above. The resource constraints faced by the Government in this period t are shown in Equation 20:


(20) $$F(K_{t},N_{t})+K_{t}=L_{t}C_{1,t}+L_{t-1}C_{2,t}+K_{t+1}$$

Given the initial conditions and resource constraints, the government maximizes the social welfare function by selecting the consumption of young and old individuals and the capital stock at the beginning of the next period, and solves the social welfare function by constructing the Lagrange function to obtain the expression of capital per worker as follows:


(21) $$k^{*}={\left(\frac{n^{*}-\rho }{\mathrm{\rho A\alpha}}\right)}^{\frac{1}{\alpha -1}}$$

By combining the level of capital per worker under the maximization of social welfare and the level of capital per worker under the equilibrium state we can obtain the optimal PERS immediately when the economy is in a steady state. By combining Equation 16 and Equation 21, we get:


(22) $$\begin{array}{l} \delta =\frac{\begin{array}{l} \mathrm{z\rho \beta}(\alpha -1+\tau -\alpha \tau )+n\{-\mathrm{\beta z}(\alpha -1)-\rho [x+\\ \beta +\mathrm{x\alpha \beta}+\alpha (z-\beta +\mathrm{\beta z})]+\tau (\alpha -1)(\mathrm{z\beta}-\rho \beta +\mathrm{z\rho})\} \end{array}}{{\left(n*\right)}^{2}\mu (\alpha -1)(n^{*}\beta +\rho -\rho \beta )}\\ +\frac{\begin{array}{l} {\left(n*\right)}^{3}[\alpha \beta \mu -\beta \mu +\mathrm{v\beta}(\alpha +\alpha \rho -\alpha \tau -1+\tau )+\mathrm{v\rho}\\ \left(\alpha +\tau -\alpha \tau )\right] \end{array}}{{\left(n*\right)}^{2}\mu (\alpha -1)(n^{*}\beta +\rho -\rho \beta )}\\ +\frac{\begin{array}{l} {\left(n*\right)}^{2}\{\alpha \rho (\tau -1)-\rho \tau +\beta [1-\alpha +\rho (v+\mu )-\alpha \rho \\ \left(1+v+\mu )+\tau (\alpha -1)(1+\mathrm{v\rho})]\right\} \end{array}}{{\left(n*\right)}^{2}\mu (\alpha -1)(n^{*}\beta +\rho -\rho \beta )} \end{array}$$

From Equation 22, the discount factor of utility in old age β , the social security contribution rate τ , the TRS z , the time needed for young people to raise a unit of children v , the elasticity of capital output α , the birth rate of the population $n^{*}$ , the economic cost needed for young people to raise a unit of children μ , the social discount factor ρ , and the time of delayed retirement x , and so on, all affect the optimal PERS. When the economy is in the equilibrium state, both capital per worker and the birth rate converge to constants, and the economic growth rate is equal to the birth rate, which is also a constant, so the comprehensive technical level A does not affect the optimal level of the PERS δ .

## 4 Numerical simulation and analysis

### 4.1 Parameters

Time investment in raising children per unit v ,. Referring to the approach of Yan (15), this paper sets the time investment of young people in raising a child at 0.2.

TRS z . Due to data limitations, this paper sets to 0.2. To verify the robustness of changing the value of the parameter, z=0.1,z=0.3 is used for robustness testing in this paper.

The proportion of the cost of raising a child per unit to wages μ . Based on the research of Liao (22) and in consideration of the current situation in China, this paper sets the proportion of the cost of raising a child per unit to wages at 0.2.

Capital output elasticity α . In developed countries such as the United States, the capital output elasticity is 0.3. Given that China is still a developing country, this paper refers to Zhou et al. (23) and sets the capital output elasticity α at 0.25.

The birth rate n . According to the “National Population Development Plan (2016–2030),” this paper sets the birth rate at 1.5.

Social security contribution rate τ . According to the “Decision of the State Council on Improving the Basic Pension Insurance System for Enterprise Employees,” this paper sets the social security contribution rate at 0.16.

Discount factor for older individual’s utility β . This paper refers to Jing and Hu (24) and sets the discount factor for older individual’s utility at 0.89.

Degree of preference for children θ . Referring to the parameter settings of Fanti and Gori (25), this paper sets the degree of preference for children of a representative individual at 0.4.

Social discount factor ρ . The social discount factor reflects the government’s consideration of the utility of different generations. Feng and Han (1) showed that the social discount rate is to some extent related to the economic growth rate. This paper assumes the social discount factor is equal to the actual economic growth rate. Assuming that the current average nominal economic growth rate in China is 5.5% and the average inflation rate is 3%, the social discount factor is calculated to be 0.43 (see Table 1).

**Table 1:** Parameter calibration.

*v*	*z*	*µ*	*α*	*n*	*τ*	*β*	*θ*	*ρ*
0.2	0.2	0.2	0.25	1.5	0.16	0.89	0.4	0.43

Time of delayed retirement x . According to China’s current retirement policy, the initial retirement age is set at 55. Based on the “gradual” delayed retirement policy, when the retirement age is between 55 and 60, the values of x are calculated as [1/30, 1/15, 1/10, 2/15, 1/6].

### 4.2 The impact of delayed retirement on the optimal PERS

**Figure 1:**
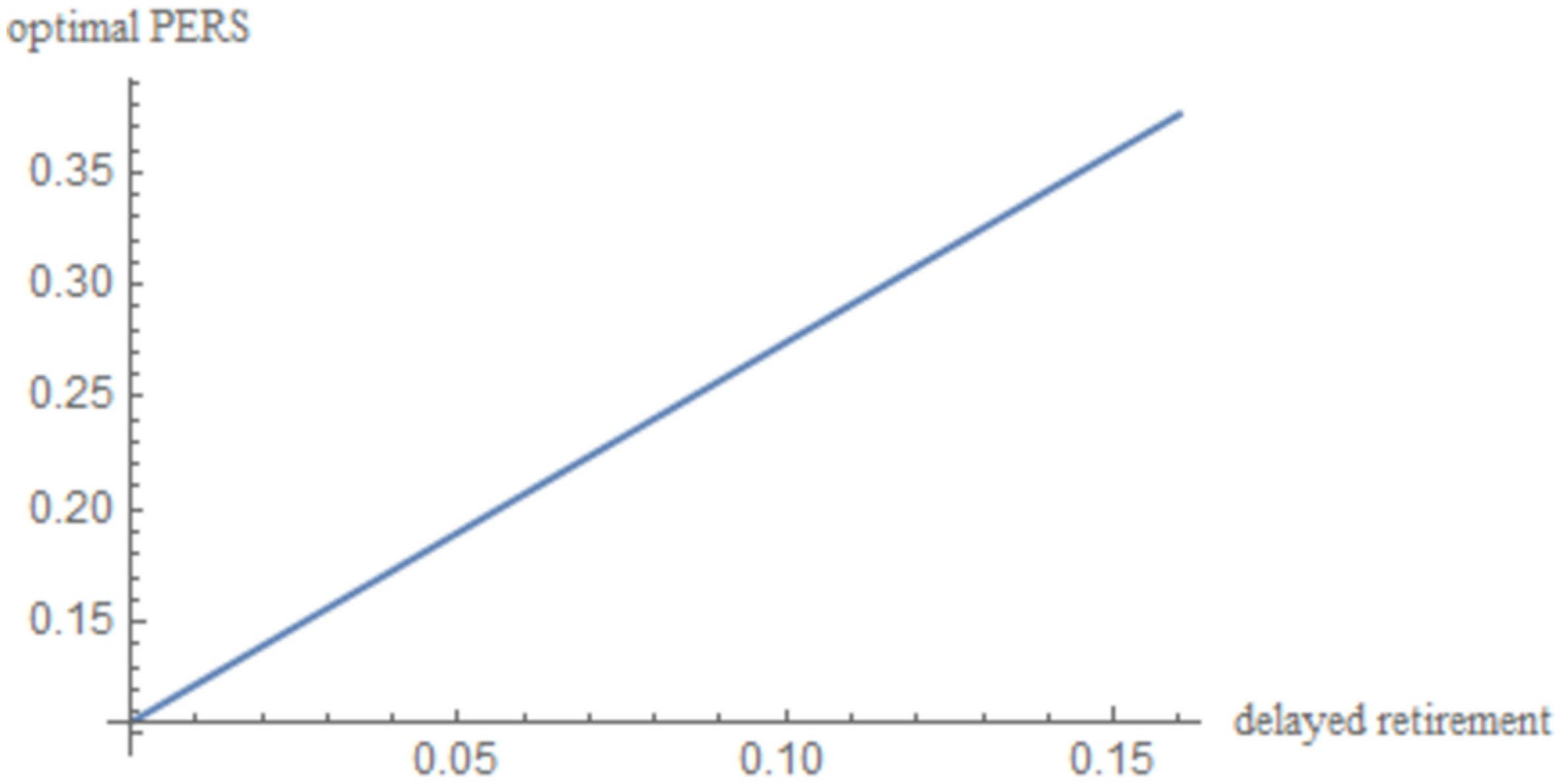
The impact of delayed retirement on the optimal PERS.

To examine the relationship between the optimal PERS and the duration of delayed retirement under the maximization of social welfare, this paper substitutes the discount factor for older individual’s utility β , the social security contribution rate τ , the TRS z , the time investment in raising a child per unit v , the capital output elasticity α , the birth rate $n^{*}$ , the proportion of the cost of raising a child per unit to wages μ , and the social discount factor ρ into Equation 22. By incorporating these parameters into Equation 22, we obtain the simulation results, which are illustrated in Figure 1 and reported in Table 2.

**Table 2:** The optimal PERS under different population birth rates and varying retirement delay durations.

Delayed retirement	*n* = 1.4	*n* = 1.5	*n* = 1.6
x=0	0.072	0.105	0.141
x=1/30	0.137	0.162	0.191
x=1/15	0.202	0.218	0.241
x=1/10	0.266	0.274	0.29
x=2/15	0.331	0.331	0.339
x=1/6	0.395	0.387	0.389

Figure 1 shows a positive correlation between the optimal PERS and the time of delayed retirement under the maximization of social welfare. This can be explained from three perspectives:

First, delayed retirement allows older adults to earn wages in old age, which are generally higher than pension benefits. This provides the older individuals with the financial capacity to contribute more to the PERS. The older individuals also can gain pleasure and rewards from providing the ERS, including emotional satisfaction from their grandchildren and material reciprocation from the younger generation.

Second, increasing the PERS can reduce the financial pressure and burden on young people, allowing them to focus more on their work and earn higher incomes. The collaboration between the older individuals and the young maximizes the overall welfare of the family.

Finally, influenced by traditional Chinese ethics and values, the older individuals regard it as an obligation to provide grandparental caregiving to their grandchildren as. Yan (15) shows that delayed retirement postpones the retirement point for the older individuals, reducing the TRS. Since delayed retirement limits TRS, the older individuals will provide economic compensation, thereby increasing PERS.

### 4.3 Solving for the optimal PERS

Considering the marginal cost of raising children increases with the number of children and that there is a cumulative effect of the marginal cost of raising children, this paper will calculate the optimal PERS under different population birth rates and varying retirement delay durations.

Table 2 presents the optimal PERS corresponding to changes in retirement age under different population birth rates. Based on the numerical simulation results, it can be observed that regardless of changes in the population birth rate, the optimal PERS shows an upward trend with the increase of the delayed retirement time. When n=1.5 , the optimal PERS is 10.5% without delayed retirement, which is at a relatively low level. With each additional year of delayed retirement, the optimal PERS increases by approximately 5.6%. When the delay in retirement reaches 5 years, the optimal PERS rises to 38.7%, indicating that delayed retirement has a significant impact on the older adults decision-making regarding grandparental caregiving.

First, this is because delayed retirement allows the older individuals to earn a higher wage than their pension, and the old-age wage plus savings from their younger years can satisfy the utility of their individual consumption in old age. In pursuit of maximizing family utility, the older individuals will allocate part of their income to the upbringing of their grandchildren. As shown in Table 2, regardless of the changes in the length of retirement delay, the optimal PERS generally increases further as the birth rate rises. This is because there is a cumulative effect on the marginal cost of raising children. As the number of children increases, the financial burden on young parents becomes heavier, and out of love for their own children, the older individuals is compelled to increase their PERS. Therefore, delayed retirement has a positive impact on the participation rate in economic grandparental caregiving.

### 4.4 The relationship between the delayed retirement, TRS and optimal PERS

**Figure 2:**
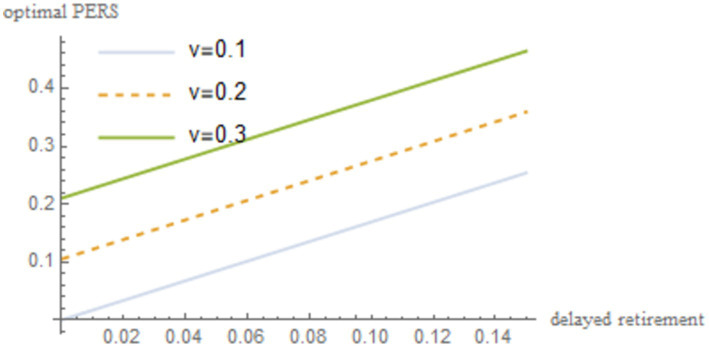
The relationship between delayed retirement, TRS, and optimal PERS.

To investigate the relationship among delayed retirement, TRS and optimal PERS under social welfare maximization, the discount factor for older individual’s utility β , the social security contribution rate τ , the time investment in raising a child per unit v , the capital output elasticity α , the birth rate $n^{*}$ , the proportion of the cost of raising a child per unit to wages μ , and the social discount factor ρ are incorporated into Equation 22. The resulting outcomes are illustrated in Figure 2.

As shown in Figure 2, under the condition of maximizing social welfare, a moderate delay in retirement increases both the TRS and optimal PERS. However, there is a negative correlation between the TRS and optimal PERS. Delayed retirement reduces the TRS, but in order to gain emotional satisfaction from their grandchildren, the older individuals will provide economic compensation. To some extent, there is a negative correlation between the TRS and optimal PERS. For the older individuals to increase the PERS, they need sufficient economic resources. By spending some time working, the older individuals can increase their income, thereby increasing their PERS.

### 4.5 The impact of delayed retirement and the optimal PERS on social welfare

**Figure 3:**
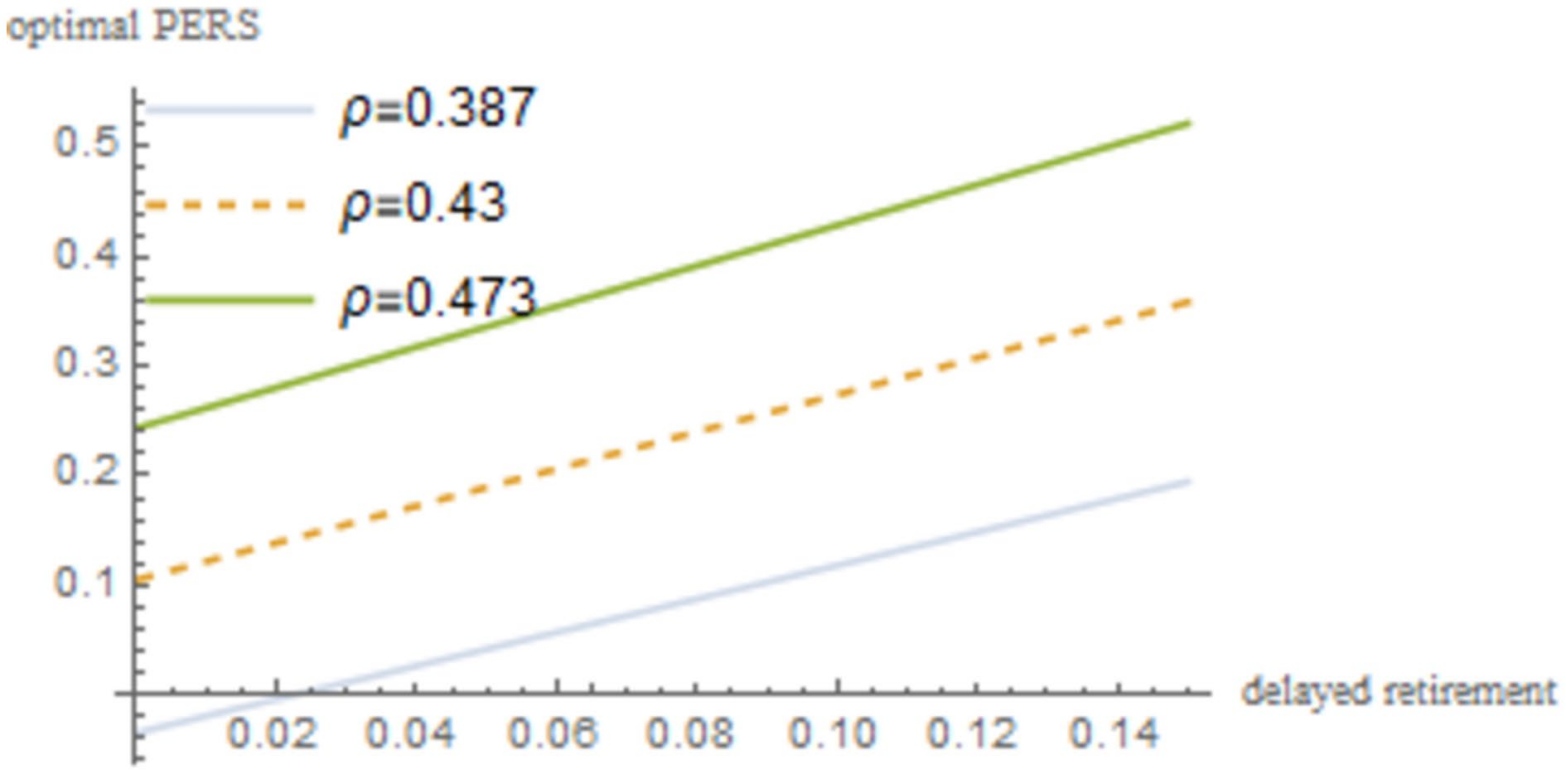
The relationship between delayed retirement, optimal PERS, and social welfare.

By simultaneously solving Equations 4, 5, 9–11, 14, 19, and incorporating the discount factor for older individual’s utility β , the social security contribution rate τ , the TRS z , the time investment in raising a child per unit v , the capital output elasticity α , the birth rate $n^{*}$ , the proportion of the cost of raising a child per unit to wages μ , and the social discount factor ρ , the effects of delayed retirement and the optimal PERS on social welfare are derived. The results are presented in Figure 3.

As shown in Figure 3, delayed retirement can increase social welfare to some extent. It allows older adults to earn wages higher than pension benefits, thereby enhancing consumption utility in both youth and old age. At the same time, it may reduce the birth rate and thus lower the utility derived from grandchildren. This paper shows that the positive impact of delayed retirement on social welfare outweighs its negative impact.

The optimal PERS increases social welfare, which can be explained from three perspectives. First, the increase in the optimal PERS reduces the economic burden on young people, meaning they have more money to spend on consumption, thereby increasing their consumption utility and having a positive effect on social welfare. Second, while the increase in the optimal PERS reduces the older adults own consumption utility, which has a negative effect on social welfare. Finally, the optimal PERS increases the birth rate, increases the utility the older individuals derives from their grandchildren, and has a positive impact on social welfare. Therefore, the optimal PERS can increase social welfare.

### 4.6 Robustness test

**Figure 4:**
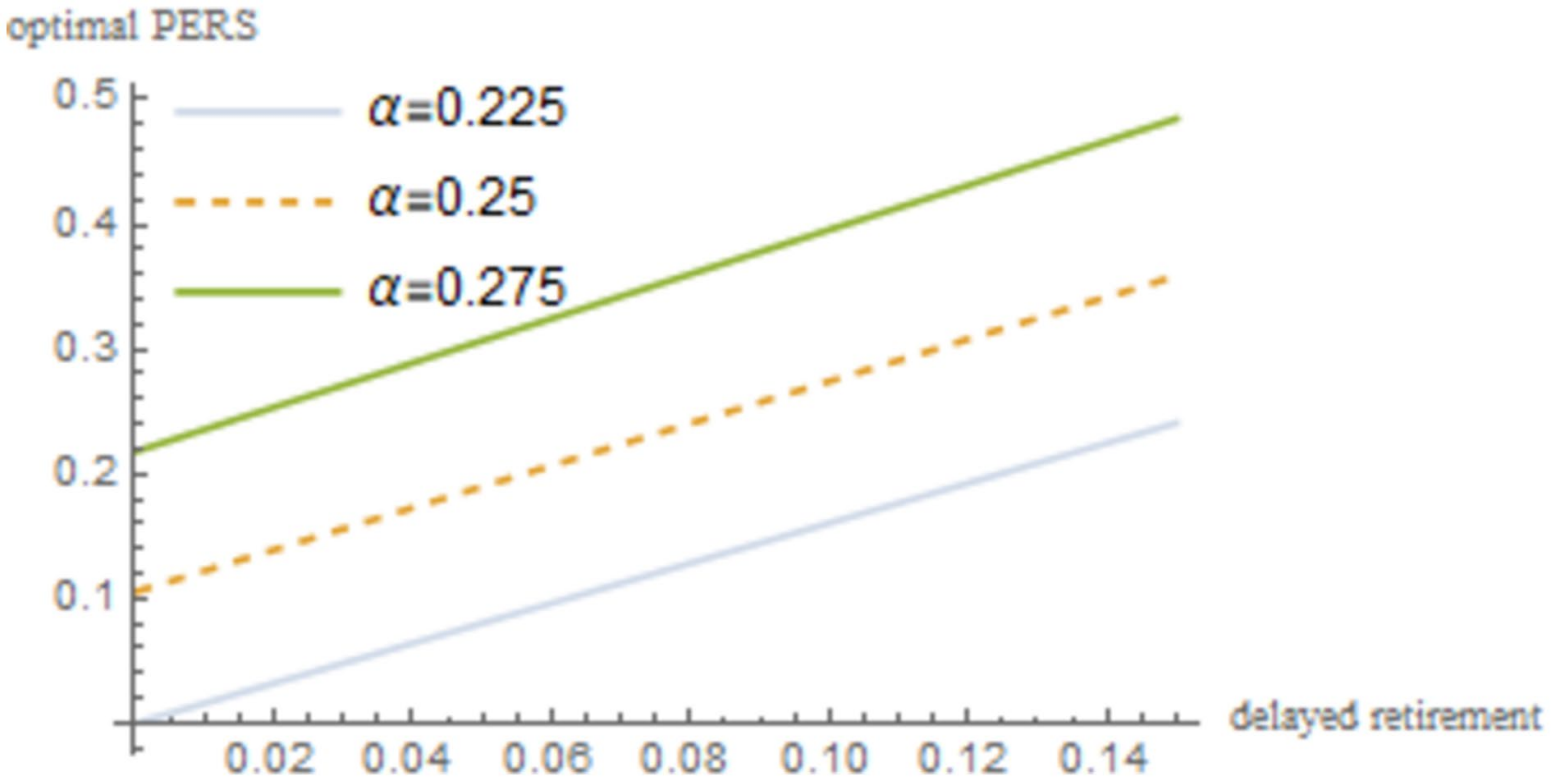
Sensitivity analysis results of *v*.

**Figure 5:**
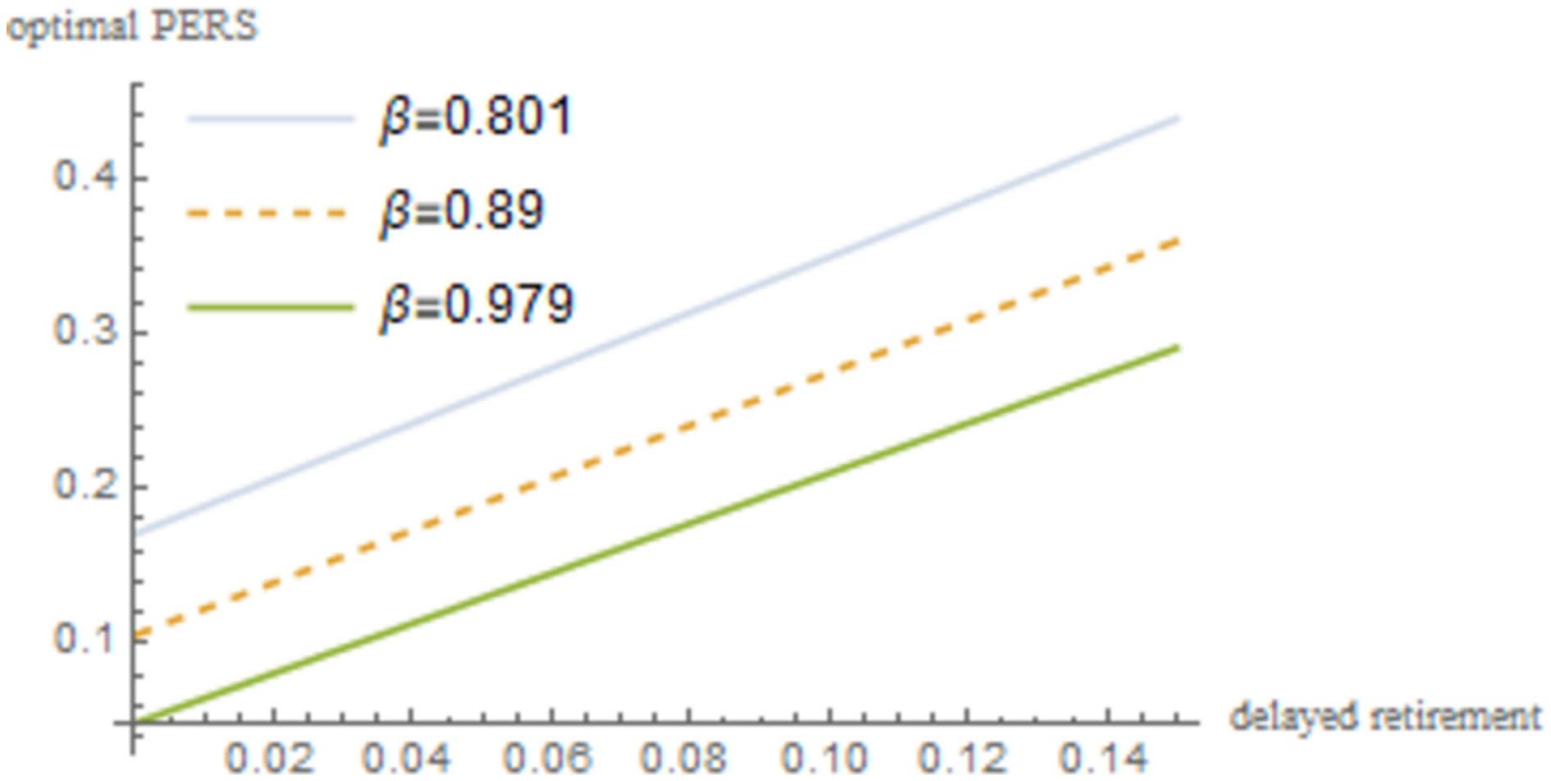
Sensitivity analysis results of *z*.

**Figure 6:**
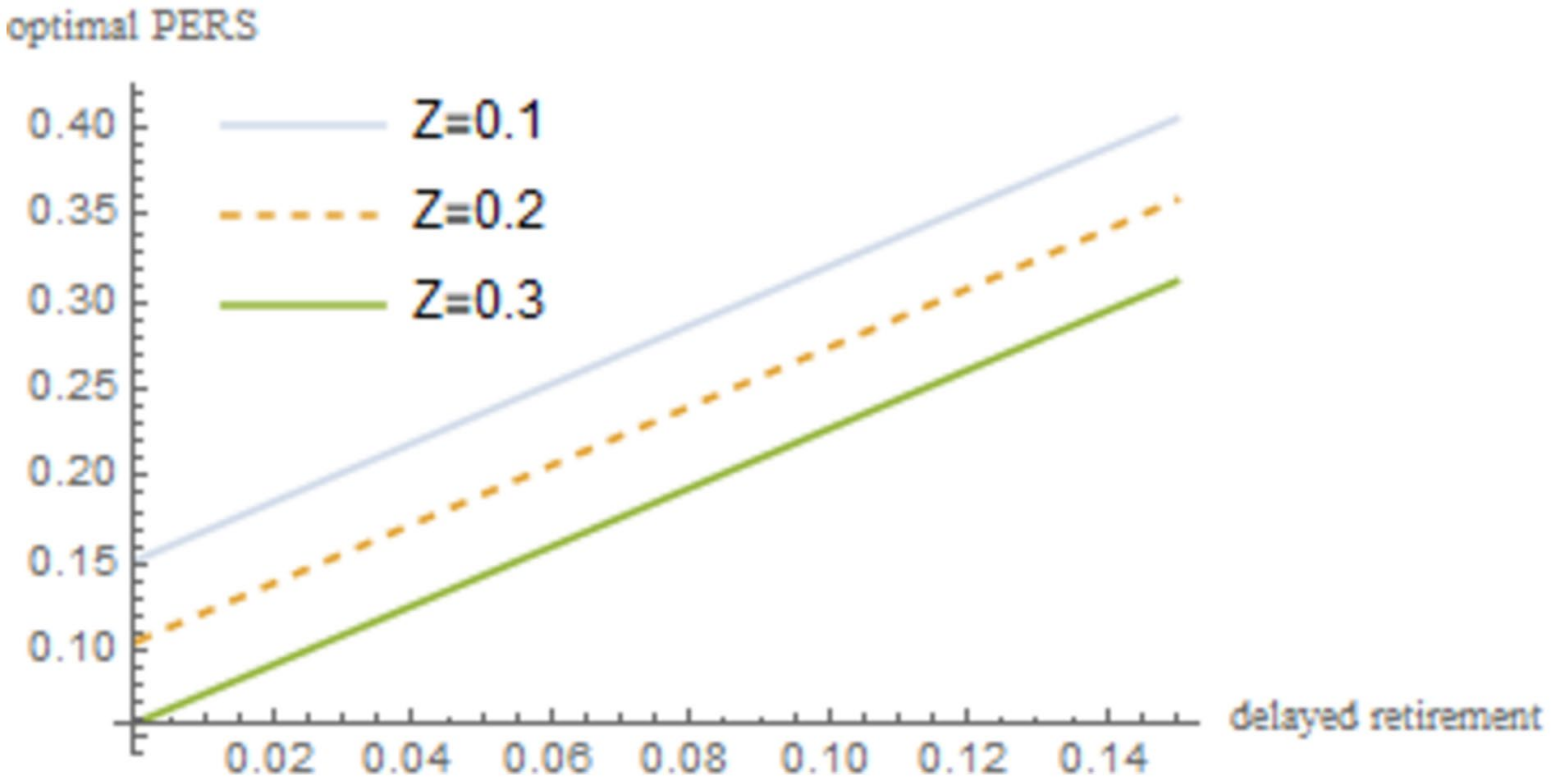
Sensitivity analysis results of *α*.

**Figure 7:**
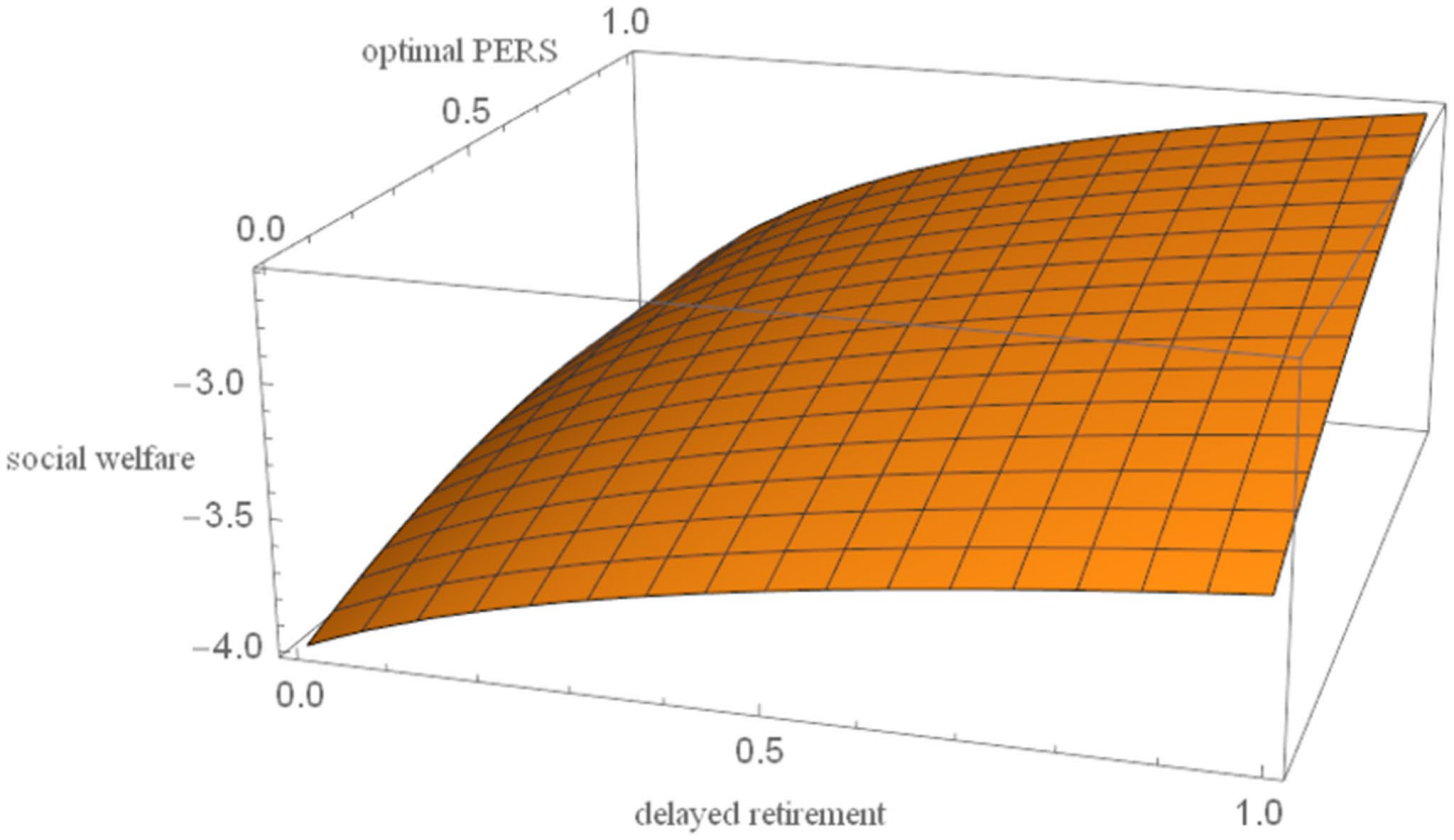
Sensitivity analysis results of *ρ*.

**Figure 8:**
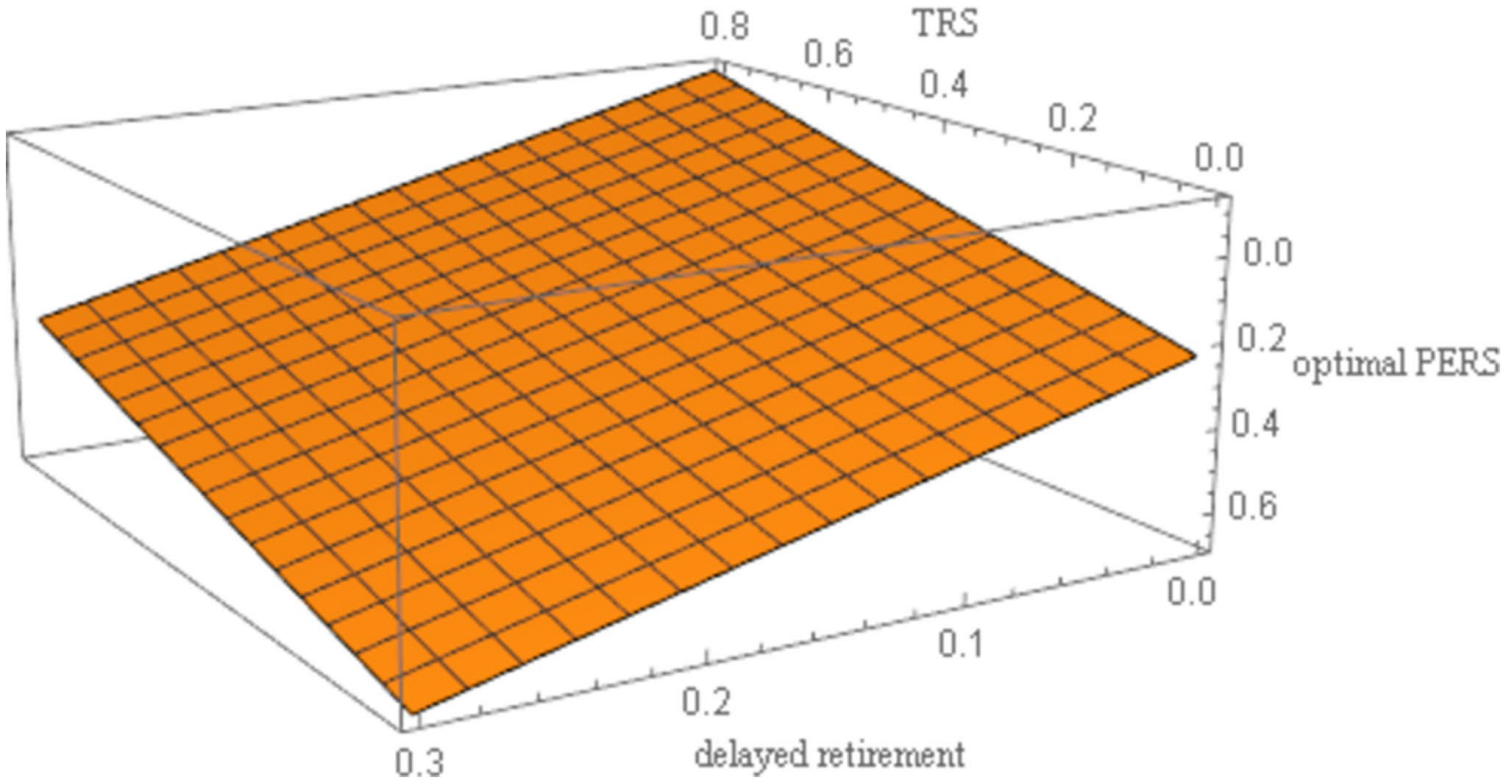
Sensitivity analysis results of *β*.

To verify that the impact of delayed retirement on the optimal PERS is not a specific result obtained under particular parameters, this paper conducts a sensitivity analysis on the time investment in raising a child per unit v , the TRS z , capital output elasticity α , social discount factor ρ , and the discount factor for older individual’s utility β . The robustness of the results is shown in Figures 4–8.

As shown in Figures 4–8, changes in the time investment in raising a child per unit, the TRS, capital output elasticity, social discount factor, and the discount factor for older individual’s utility do not significantly alter the direction or magnitude of the impact of delayed retirement on the optimal PERS. This confirms the robustness of the results in Figure 1.

The results in Table 2 were calculated based on the benchmark parameters. Considering that the reasonableness of the benchmark parameters can affect the optimal PERS, this paper conducts a sensitivity analysis on the following benchmark parameters to verify the robustness of the results in Table 2: time investment in raising a child per unit v , TRS z , capital output elasticity α , social discount factor ρ , and discount factor for older individual’s utility β . To do this, we analyze the optimal PERS when the population birth rate is n=1.5 and the retirement age is between 55 and 60. The results are shown in Table 3.

**Table 3:** Sensitivity analysis of the optimal PERS.

Parameters		x=0	x=1/30	x=1/15	x=1/10	x=2/15	x=1/6
v	0.1	0.011	0.057	0.113	0.169	0.226	0.282
	0.3	0.21	0.266	0.323	0.379	0.436	0.492
z	0.1	0.152	0.208	0.265	0.321	0.377	0.434
	0.3	0.059	0.115	0.171	0.228	0.284	0.34
α	0.225	0.001	0.054	0.107	0.161	0.214	0.268
	0.275	0.218	0.277	0.337	0.396	0.455	0.515
β	0.801	0.171	0.231	0.289	0.349	0.408	0.468
	0.979	0.049	0.102	0.156	0.21	0.263	0.317
ρ	0.387	NA	0.017	0.068	0.119	0.169	0.22
	0.473	0.244	0.306	0.367	0.429	0.491	0.553

For ease of calculation and comparison, the values of v , z , α , β , ρ are set to fluctuate by ±10%. Considering that the optimal PERS cannot be negative, model results with negative values are not shown.

From Table 3, it can be observed that the time investment in raising a child per unit v is positively correlated with the optimal PERS. A higher v means that young people need to spend more time raising children, which reduces their working hours and income. In response, the older individuals increases PERS to maximize family utility. The TRS z is inversely related to the optimal PERS. On one hand, longer caregiving time reduces the time the older individuals can spend working, thus decreasing their income. On the other hand, it gives young people more time to work, providing them with enough income to raise children.

A higher capital output elasticity α implies a higher return on capital for the older individuals, leading to higher disposable income and an increased PERS. Therefore, capital output elasticity α is positively correlated with the optimal PERS. A higher discount factor for older individual’s utility β indicates that representative individuals place more importance on their post-retirement standard of living and consumption utility. To maintain their utility level, the older individuals may reduce the optimal PERS.

The social discount factor ρ reflects the government’s emphasis on the utility of young people. An increase in PERS can effectively enhance the utility of the younger generation, so the social discount factor ρ has a positive correlation with the optimal PERS.

In conclusion, the optimal PERS is influenced by parameters such as the time investment in raising a child per unit v , the TRS z , capital output elasticity α , the social discount factor ρ , and the discount factor for older individual’s utility β . Therefore, when solving for the optimal PERS, it is important to ensure that the values of the benchmark parameters align with the current economic conditions.

## 5 Further analysis

The above analysis in this paper is based on the pay-as-you-go social security system, primarily for the following two reasons:

First, with the increasing aging of population, the imbalance between the revenue and expenditure in China’s pay-as-you-go system social security fund has become increasingly prominent, and the issue of “empty account” has garnered significant attention from scholars.

Second, there are significant differences in the use of social security income under the pay-as-you-go system and the fully funded pension system. Under the pay-as-you-go system, the social security funds paid by young people at the current time are distributed to the older individuals who retire at the current time; under the fully funded pension system, the social security funds paid by young people are like savings, and they will receive the principal of the social security account and corresponding interest when they retire.

The impact of delayed retirement and PERS on the birth rate may differ across social security systems. Therefore, this paper will explore the impact of delayed retirement and the PERS on the birth rate under the fully funded pension system.

Assuming that under the fully funded pension system, the social security funds paid by young people are used for investment, the fund revenue and expenditure equation is as follows:


(23) $$w_{t}\tau [1-({\mathrm{vn}}_{t}-\frac{z_{t}}{n_{t-1}})]R_{t+1}L_{t}+\tau w_{t+1}{\mathrm{xL}}_{t}=(1-x)P_{t+1}L_{t}$$

The left side of the equation is the total income of the social security fund, $w_{t}\tau [1-({\mathrm{vn}}_{t}-\frac{z_{t}}{n_{t-1}})]L_{t}R_{t+1}$ is the principal and interest of the social security fund paid by the older individuals when they were young, $\tau w_{t+1}{\mathrm{xL}}_{t}$ is the social security fund paid by the older individuals who delayed retirement, and the right side of the equation is the total expenditure of the social security fund. Dividing both sides of Equation 23 by $L_{t}$ , we get:


(24) $$w_{t}\tau [1-({\mathrm{vn}}_{t}-\frac{z_{t}}{n_{t-1}})]R_{t+1}+w_{t+1}\mathrm{x\tau}=(1-x)P_{t+1}$$

Under the fully funded pension system, with a cleared capital market, capital accumulation comes from representative individuals’ savings and social security contributions, all of which are invested in the next period. The dynamic equation of capital accumulation is:


(25) $$K_{t+1}=S_{t}L_{t}+w_{t}\tau [1-({\mathrm{vn}}_{t}-\frac{z_{t}}{n_{t-1}})]L_{t}$$

Divide both sides of Equation 25 by the total labor $N_{t+1}$ of t+1 , obtain the t+1 average labor capital $k_{t+1}$ see Equation 26 for details:


(26) $$\begin{array}{l} k_{t+1}=\frac{K_{t+1}}{[1-(vn_{t+1}-\frac{z_{t+1}}{n_{t}})]L_{t+1}+{\mathrm{xL}}_{t}}\\ =\frac{S_{t}+w_{t}\tau [1-({\mathrm{vn}}_{t}-\frac{z_{t}}{n_{t-1}})]}{[1-(vn_{t+1}-\frac{z_{t+1}}{n_{t}})]n_{t}+x} \end{array}$$

By substituting Equations 6, 9, 10, 24 into (26) and combining $k_{t+1}=k_{t}=k^{*}$ , $n_{t+1}=n_{t}=n^{*}$ , we can get:


(27) $${\left(k^{*}\right)}^{\alpha -1}=\frac{{\left(n^{*}\right)}^{2}\mu \delta -n^{*}x+\frac{\alpha n^{*}(n^{*}-{\left(n^{*}\right)}^{2}v+x+z)(1+\beta )}{\alpha -1}}{\mathrm{A\alpha \beta}{\left(n^{*}\right)}^{2}(v+\mu -\mu \delta )-\text{Aαzβ}-\mathrm{A\alpha}n^{*}\beta }$$

By substituting Equations 4, 5, 9, 10, 24 into (7) and combining $k_{t+1}=k_{t}=k^{*}$ , $n_{t+1}=n_{t}=n^{*}$ , we can get:


(28) $${\left(k^{*}\right)}^{\alpha -1}=\frac{{\left(n^{*}\right)}^{2}\delta \mu (1+\beta +\theta )-n^{*}\mathrm{\theta x}}{\begin{array}{l} \mathrm{A\alpha}n^{*}\theta +\text{Aαzθ}+\mathrm{A\alpha}{\left(n^{*}\right)}^{2}\mathrm{v\tau}(1+\beta )-\\ \;\;\;\;\;\;\mathrm{A\alpha}{\left(n^{*}\right)}^{2}(1+\beta +\theta )(v+\mu -\delta \mu ) \end{array}}$$

To explore the impact of delayed retirement and the PERS on the equilibrium population birth rate, define the right-hand side of Equation 27 as $\psi (x,\delta ,n^{*})$ , and the right-hand side of Equation 28 as $\varpi (x,\delta ,n^{*})$ . Then, we have $\Omega (x,\delta ,n^{*})=\varpi (x,\delta ,n^{*})-\psi (x,\delta ,n^{*})=0$ . According to the implicit function theorem, we can obtain (Equations 29 and 30):


(29) $$\frac{dn^{*}}{\mathrm{dx}}=-\frac{\Omega_{x}(x,\delta ,n^{*})}{\Omega_{n^{*}}(x,\delta ,n^{*})}=-\frac{\varpi_{x}(x,\delta ,n^{*})-\psi_{x}(x,\delta ,n^{*})}{\varpi_{n^{*}}(x,\delta ,n^{*})-\psi_{n^{*}}(x,\delta ,n^{*})}$$

(30) $$\frac{dn^{*}}{\mathrm{d\delta}}=-\frac{\Omega_{\delta }(x,\delta ,n^{*})}{\Omega_{n^{*}}(x,\delta ,n^{*})}=-\frac{\varpi_{\delta }(x,\delta ,n^{*})-\psi_{\delta }(x,\delta ,n^{*})}{\varpi_{n^{*}}(x,\delta ,n^{*})-\psi_{n^{*}}(x,\delta ,n^{*})}$$

As calculated, $\mathrm{sign}(dn^{*}/\mathrm{dx})<0$ , indicating that delayed retirement will reduce the birth rate in the equilibrium state, $\mathrm{sign}(dn^{*}/\mathrm{d\delta})>0$ , shows that increasing the PERS will raise the birth rate in the equilibrium state, which is consistent with the previous conclusion.

## 6 Discussion

This paper is based on the OLG model that includes grandparental caregiving and delayed retirement, and conducts research using China as a case study. Its core conclusions are not only applicable to the Chinese context, but also have universal implications for countries facing the dual challenges of population aging and low fertility rates worldwide. Research has found that the delayed retirement policy affects the birth rate through a dual path of “reduced TRS” and “increased PERS,” with the negative effect of the former outweighing the positive effect of the latter, ultimately leading to a decrease in birth rate. This mechanism is common in most aging countries around the world: In both developed and developing countries, delayed retirement increases older adults’ labor participation and reduces their ability to provide time support for childcare—raising the time cost of childrearing, a key factor suppressing fertility. At the same time, higher income strengthens their capacity to provide economic support. Although this positive effect varies in strength in different countries, it is generally present in various economies. Therefore, the “PERS-TRS substitution effect” revealed by the model in this paper can provide a unified analytical framework for understanding the birth rate side effects of delayed retirement policies worldwide.

Furthermore, this paper reaches a consistent conclusion under both the pay-as-you-go and full accumulation social security frameworks, indicating that the impact of delayed retirement on birth rate is not a product of a specific social security system, but rather an inevitable result of the interaction between population structure transformation and labor market policies. For countries dominated by the pay-as-you-go system, although delayed retirement can alleviate the pressure of pension payments, we need to be wary of its suppression of the birth rate, which may exacerbate the vicious cycle of “decreasing payment population”; For countries that rely on the fund accumulation system, although delayed retirement increases the accumulation of personal pension accounts, the positive incentive of PERS is still difficult to offset the negative effect of reduced TRS. This means that the “birth cost” of delayed retirement policies is an implicit cost that needs to be considered jointly in policy formulation by all countries.

At the level of policy implications, this paper’s suggestion on “matching supporting policies with delayed retirement” has global applicability. In response to the inhibitory effect of delayed retirement on birth rate, countries can introduce differentiated measures based on their own national conditions, essentially balancing policy contradictions through “social support replacing family support”; The model shows that delayed retirement can improve overall social welfare, but its gain depends on the positive effect of the PERS in the economy. This suggests that countries need to pay attention to intergenerational resource allocation fairness and provide flexible retirement options for special groups to avoid worsening welfare losses; although there are differences in the forms of grandparental caregiving under different cultural backgrounds, the substitution relationship between TRS and PERS is universal. Countries can optimize the structure of grandparental caregiving through policy guidance and maximize the comprehensive benefits of policies.

In addition to the economic mechanisms highlighted in this paper, it is also important to incorporate perspectives from public health, sociology, and social policy. From a public health perspective, delayed retirement not only alters the intergenerational allocation of caregiving resources but also affects the mental and physical health of both the older individuals and younger parents, as caregiving stress and work–family conflicts may exacerbate health risks. From a sociological perspective, family structures, gender roles, and cultural norms shape the extent to which grandparents participate in childrearing; in particular, the burden of caregiving often falls disproportionately on women, suggesting that delayed retirement policies may have heterogeneous effects across gender and household types. From a social policy perspective, the findings imply that complementary policies are needed to mitigate the unintended fertility-suppressing effects of delayed retirement—for example, expanding affordable childcare services, enhancing community-based eldercare and childcare integration, and providing parental leave policies that reduce the time cost of childrearing for young families. Integrating these broader dimensions highlights that the interaction between retirement policy and fertility behavior is not only an economic issue but also a social and public health challenge, requiring comprehensive policy coordination.

In summary, this paper not only answers the question of the birth rate impact of China’s delayed retirement policy, but also reveals the universal law of the interaction between “labor market policy-family behavior-population structure” under the background of aging, providing theoretical basis and empirical reference for countries around the world to formulate policy combinations that balance the sustainability of older individual’s care and reproductive vitality. Future research can combine the cultural, institutional, and demographic characteristics of different countries to further expand the situational adaptability of the model and provide more refined guidance for differentiated policy design.

Future research can be deepened from multiple dimensions to verify theoretical robustness and expand policy implications. Firstly, empirical tests will be conducted based on cross-border panel data, with delayed retirement intensity as the core variable, combined with the mediation effect analysis of the grandparental caregiving intensity index, to verify the negative impact of policies on birth rate and the “TRS- PERS substitution” mechanism. Grouping will be conducted according to cultural characteristics and childcare levels to reveal the heterogeneity boundaries of the effects.

Secondly, based on the refinement mechanism analysis of micro family tracking data, the impact of delayed retirement on PERS and TRS is quantified through a panel model, and the effect of grandparental caregiving on the birth rate is tested using propensity score matching method; Simultaneously utilizing natural experiments of policy shocks from Japan, South Korea, and other countries, the dynamic characteristics of policy effects are identified using the double difference method and event study method.

Thirdly, innovative research methods are used to integrate macro and micro data through mixed effects models, identify key moderating factors using machine learning, and reduce variable selection bias. The above directions can systematically verify the universality of conclusions, clarify the boundaries of policy effects, and provide precise empirical support for policy-making in aging countries worldwide.

## Data Availability

The original contributions presented in the study are included in the article, further inquiries can be directed to the corresponding author.
